# The Association of Retinopathy and Plasma Glucose and HbA1c: A Validation of Diabetes Diagnostic Criteria in a Chinese Population

**DOI:** 10.1155/2016/4034129

**Published:** 2016-10-11

**Authors:** Rui Zhang, Yufeng Li, Simin Zhang, Xiaoling Cai, Xianghai Zhou, Linong Ji

**Affiliations:** ^1^Peking University People's Hospital, Beijing, China; ^2^Beijing Pinggu Hospital, Beijing, China

## Abstract

*Aims*. This study aimed to evaluate the associations of diabetic retinopathy (DR) with fasting plasma glucose (FPG), 2-hour postload plasma glucose (2hPG), and glycated hemoglobin A1c (HbA1c) in a Chinese population.* Materials and Methods*. A total of 3124 participants, identified from a population-based survey in Pinggu district, were examined by retinal photography (45°). DR was classified according to the Early Treatment Diabetic Retinopathy Study scale. FPG, 2hPG, and HbA1c were tested and categorized by deciles, with the prevalence of DR calculated in each decile.* Results*. The prevalence of DR increased sharply in the 10th deciles, when FPG exceeded 7.03 mmol/L and HbA1c exceeded 6.4%. Analysis of the receiver operating characteristic curves showed that the optimal cutoffs for detecting DR were 6.52 mmol/L and 5.9% for FPG and HbA1c, respectively. The World Health Organization (WHO) criteria for diagnosing diabetes showed high specificity (90.5–99.5%) and low sensitivity (35.3–65.0%). Further, 6 individuals with retinopathy had normal plasma glucose; however, their characteristics did not differ from those without retinopathy.* Conclusions*. Thresholds of FPG and HbA1c for detecting DR were observed, and the WHO criteria of diagnosing diabetes were shown to have high specificity and low sensitivity in this population.

## 1. Introduction

For decades, the diagnostic criteria of diabetes mellitus were based on either the fasting plasma glucose (FPG) or 2-hour postload plasma glucose (2hPG) of the 75 g oral glucose tolerance test (OGTT). In 2010, glycated hemoglobin A1c (HbA1c) ≥ 6.5% was adopted by the American Diabetes Association [1] and was subsequently recommended by the World Health Organization (WHO) [2] for diagnosing diabetes. The establishment of these criteria originated from the observations of several cross-sectional studies [3–5]. In these studies, the thresholds of FPG, 2hPG, and HbA1c were observed above the levels at which the prevalence of diabetic retinopathy (DR), a specific complication representing the prognosis of diabetes, increased sharply. However, the specific glycemic cutoffs varied among validation studies of different ethnic groups. Further, because of ethnical differences in HbA1c [6], whether HbA1c ≥ 6.5% can appropriately reflect the changes in the risk of DR and serve as a diagnostic criterion of diabetes in the Chinese population remains unknown. Until now, only one study in China has analyzed the associations of DR and glycemic measures with similar methods to the former studies on the topic from other countries. However, this study included only participants whose FPG level was ≥5.6 mmol/L and was thus not representative of the general population.

Therefore, the aims of the present study were (1) to evaluate the associations of DR and different glycemic measurements in a general Chinese population, with the specific cutoff points estimated and (2) to test the sensitivity and specificity of the current diagnostic criteria in this population.

## 2. Materials and Methods

### 2.1. Study Population and Information Collection

A cross-sectional population-based survey for diabetes and metabolic syndrome was conducted in Pinggu district, which is located in the northeast of Beijing, from March 2012 to May 2013. A stratified random two-stage cluster sampling process was used to recruit participants. The details of the sampling method and study population have been previously described [7]. A total of 5004 individuals were invited, out of whom 3350 responded (response rate, 66.9%). After excluding 226 individuals with incomplete information of retinal photography or plasma glucose, 3124 were finally included in the current data analysis. The study was conducted with the approval of the Review Board for Clinical Research of Peking University People's Hospital Institution, and written informed consent was obtained from all participants.

A questionnaire about the medical history and physical measurements was filled out by the field researchers. Body mass index was calculated as the weight divided by height squared (kg/m^2^). The OGTT was performed in all participants without known diabetes. The details of the OGTT and biochemical measurements, including plasma glucose, HbA1c, serum alanine aminotransferase, aspartate aminotransferase, creatinine, total cholesterol, triglycerides, high-density lipoprotein cholesterol, and low-density lipoprotein cholesterol, have been previously described [7]. The fasting and 2-hour post-glucose-load serum insulin levels were measured by the electrochemical luminescence method (COBAS E411, Roche Diagnostics, Tokyo, Japan). Urine albumin and creatinine were measured using spot urine samples by immunoturbidimetric assay and Jaffe's assay (COBAS C311, Roche Diagnostics), respectively, and the urine albumin-to-creatinine ratio was calculated.

### 2.2. Ophthalmic Examination and Definition of Diabetic Retinopathy

Each participant underwent ophthalmic examination by retinal photographs taken of both eyes. Fundus photographs (45°) were taken using a “nonmydriatic” TRC NW8 fundus camera (Topcon Corporation, Tokyo, Japan), without pupil dilation, in seven fields per eye. Two qualified assessors, blinded to all participant information, graded the photographs independently. Discrepancies of the grading results between these two assessors were graded by a third ophthalmologist to confirm the final results. The level of retinopathy was defined according to the modified Early Treatment of Diabetic Retinopathy Study (ETDRS) severity scale [8]. In the present study, DR was defined as an ETDRS scale score ≥ level 20, which is defined as very mild DR. The classification was based on the grading of the worst eye.

### 2.3. Statistical Analysis

Statistical analysis was performed with SPSS for Windows 16.0 (SPSS Inc., Chicago, IL) and Stata 10.0 (Stata Corp., College Station, TX). The mean (standard deviation) value was used to describe continuous variables with a normal distribution. Categorical data are presented as a number (percentage). For other values, the median (interquartile range) values were used. Comparisons between the mean values were performed using Student *t-*tests, whereas the median values were compared using the Mann–Whitney test. For the analyses, FPG, 2hPG, and HbA1c were categorized by deciles. The Chi-square test was used to judge the differences in the prevalence of DR in each decile. Subsequently, receiver operating characteristic (ROC) curves were created for each glycemic measure to calculate and compare the areas under the curves and to find the optimal cutoffs for detecting DR by maximizing the sensitivity and specificity. The WHO criteria of diagnosing diabetes were also tested for sensitivity and specificity in this population. Finally, we compared the characteristics between those with and without retinopathy in individuals with normal plasma glucose using Student *t-*tests or the Mann–Whitney test, as appropriate.

## 3. Results

Of 3124 participants, 40 (1.3%) had DR, as defined by an ETDRS severity scale score ≥ level 20. Three individuals (0.1%) had a score ≥ level 43, which is defined as moderate nonproliferative DR. No severe nonproliferative DR or proliferative DR was discovered in the study population.

**Figure 1:**
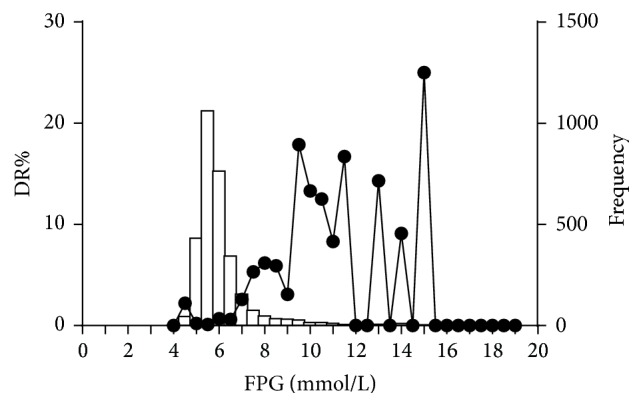
Distribution of fasting plasma glucose (FPG) and the corresponding prevalence of diabetic retinopathy (DR) in the study population.

**Figure 2:**
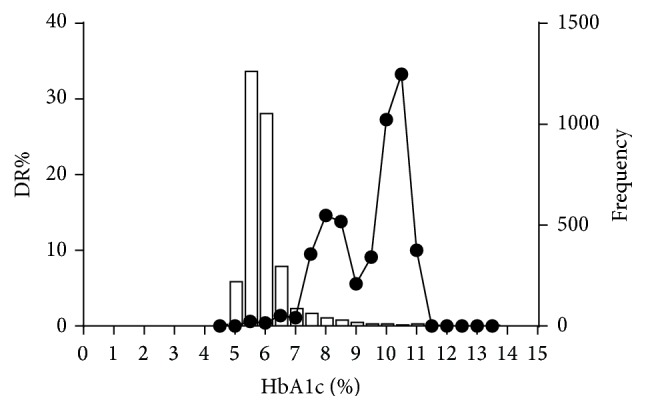
Distribution of glycated hemoglobin A1c (HbA1c) and the corresponding prevalence of diabetic retinopathy (DR) in the study population.

Table 1 shows the comparison of the characteristics of participants with and without DR. The participants with DR were older in age, with higher blood pressure, lower 2-hour post-oral-glucose-load plasma insulin level, lower serum creatinine, lower serum uric acid, and higher urine albumin-to-creatinine ratio compared to those without DR. The plasma glucose and HbA1c of the DR group were both significantly higher than in the non-DR group. Other characteristics, including sex and body mass index, were similar between the groups. Among the 40 participants with DR, 23 (57.5%) people were known diabetic patients, 7 (17.5%) were newly diagnosed with diabetes in this study, and 4 (10%) were classified as having prediabetes. Of the 23 patients with known diabetes, 13 were currently on oral antihyperglycemic drugs, 1 patient was using insulin only, and 8 received both oral drugs and insulin. The distributions of FPG and HbA1c and the corresponding prevalence of DR in this population are shown in Figures 1 and 2, respectively.

**Figure 3:**
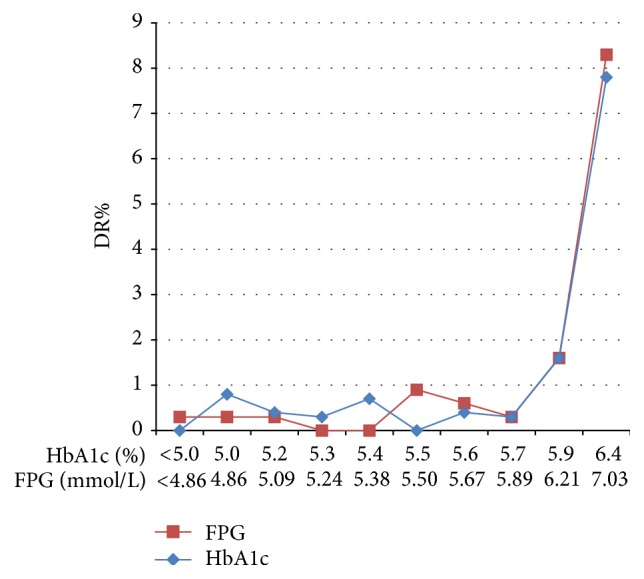
Prevalence of diabetic retinopathy (DR) according to the deciles of distribution of fasting plasma glucose (FPG) and glycated hemoglobin A1c (HbA1c) in the total 3124 subjects. The *x*-axis indicates the minimum value of each decile group.

**Figure 4:**
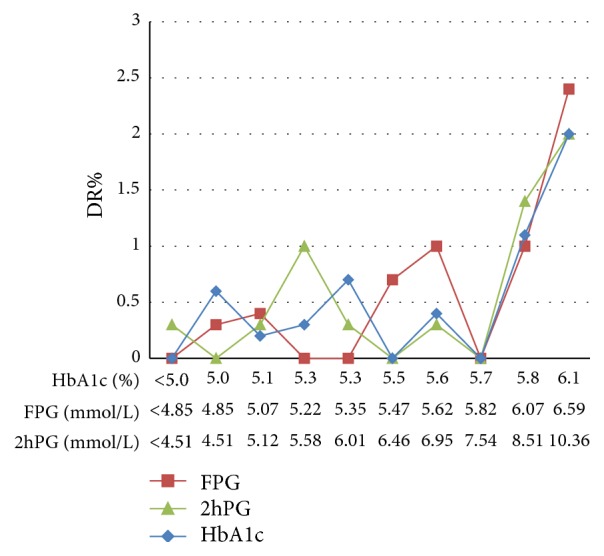
Prevalence of diabetic retinopathy (DR) according to the deciles of distribution of fasting plasma glucose (FPG), 2-hour postload plasma glucose (2hPG), and glycated hemoglobin A1c (HbA1c) in 2959 subjects after excluding those with known diabetes. The *x*-axis indicates the minimum value of each decile group.

Evidence of a threshold effect was found for the association of DR with both FPG and HbA1c (Figure 3). In the 10th deciles, the prevalence of DR increased markedly from less than 1.6% to 8.3% and 7.8%, with minimum levels of 7.03 mmol/L for FPG and 6.4% for HbA1c (*p* < 0.01). However, after excluding 165 patients with known diabetes, no distinct threshold value existed for FPG, 2hPG, or HbA1c (Figure 4).

**Figure 5:**
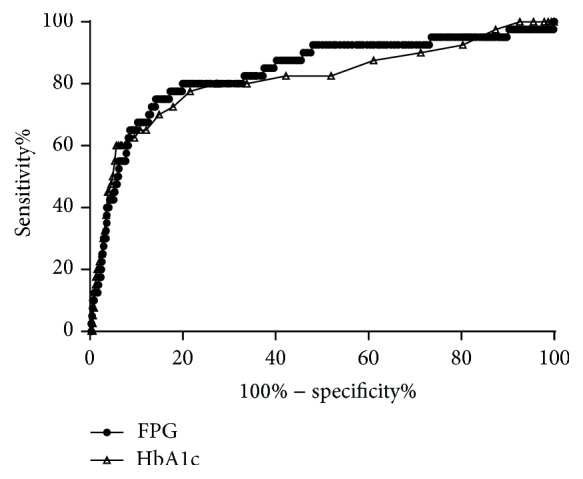
Receiver operating characteristic curves of glycated hemoglobin A1c (HbA1c) and fasting plasma glucose (FPG) for detecting diabetic retinopathy in the total 3124 subjects.

**Figure 6:**
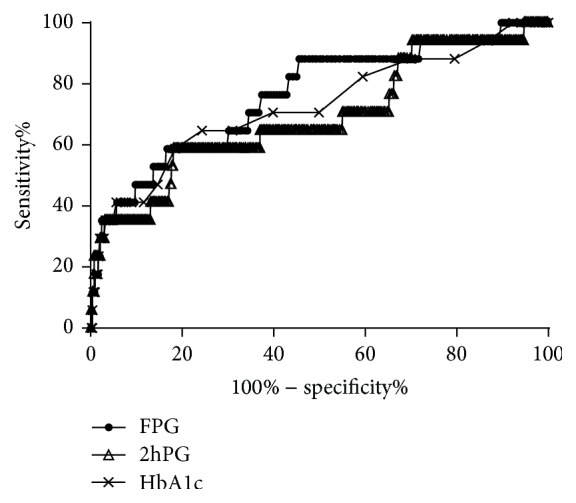
Receiver operating characteristic curves of glycated hemoglobin A1c (HbA1c), fasting plasma glucose (FPG), and 2-hour postload plasma glucose (2hPG) for detecting diabetic retinopathy in 2959 subjects after excluding those with known diabetes.

In the analysis of the ROC curves, the areas under the ROC curves for FPG and HbA1c were 83.7% (95% confidence interval [CI] 76.2, 91.2) and 81.4% (95% CI 73.0, 89.7), respectively, with no significant difference in the ability to predict DR (*p* = 0.534) (Figure 5). After excluding patients with known diabetes, the areas of all three measures were diminished to 76% (95% CI 63.6, 88.4) for FPG, 72.7% (95% CI 58.6, 86.9) for HbA1c, and 68.7% (95% CI 54.1, 83.4) for 2hPG (Figure 6). The areas of the three measures were not significantly different. The optimal cutoff points, determined by maximizing the sensitivity and specificity of FPG and HbA1c using the Youden index, were 6.52 mmol/L and 5.9%, respectively, in the total 3124 participants (Table 2).

The sensitivity and specificity of the different cutoff points for detecting DR derived from the distribution of the deciles and ROC curves in the present study, as well as the current WHO diagnostic criteria, are compared in Table 2. The American Diabetes Association recommendation for diagnosing diabetes of HbA1c ≥6.5% showed a similar sensitivity and a higher specificity as the cutoff of 6.4% identified herein for detecting DR in this population. The FPG level of ≥7.03 mmol/L for diagnosing diabetes in the present study was slightly superior to the WHO criteria in specificity, with similar sensitivity (Table 2). The overall performance of the WHO criteria revealed good specificity (90.5%, 91.7%, and 99.5%) but low sensitivity (65.0%, 35.3%, and 62.5%) in this population.

In addition, among the 40 individuals with DR, 6 (15%) had completely normal FPG and 2hPG levels, and 5 of these individuals had an FPG < 5.6 mmol/L (Figure 1); all of them were classified as having mild DR. Compared to the other participants with normal glucose levels, these 6 individuals did not differ in any of the demographic characteristics or possible risk factors of DR such as HbA1c and hypertension.

## 4. Discussion

In this sample of a Chinese population living in one district in Beijing, thresholds of FPG and HbA1c for detecting DR were observed between the ninth and tenth deciles (FPG exceeding 7.03 mmol/L and HbA1c exceeding 6.4%). After excluding those with known diabetes, the threshold effect was not overt for FPG, 2hPG, or HbA1c. The optimal cutoffs, as determined by ROC curve analysis, were 6.52 mmol/L for FPG and 5.9% for HbA1c. Further, we found that the current diagnostic criteria showed high specificity and low sensitivity in this population.

Previous studies [3–5] on the associations between glycemic measures and DR have made important contributions to the establishment of the current criteria [9, 10] for diagnosing diabetes. However, the specific cutoff points varied considerably between these studies, and the early studies had the common limitation of imprecise assessment of DR by either direct clinical ophthalmoscopic examination or single-field retinal photography. In recent years, several studies [11–18] in various ethnic groups have tried to reevaluate the diagnostic criteria and to determine the most accurate cutoff points. However, the results are still relatively inconsistent. Some of these previous studies [14] reported a continuous association of DR with fasting plasma glucose and HbA1c, without a clear glycemic threshold. However, most other studies support the existence of a glycemic threshold for the prevalence of DR, with the specific cutoff ranging from 5.8 to 7.5 mmol/L for FPG and 5.3–6.8% for HbA1c. Among these studies, a large data-pooling analysis involving approximately 45,000 participants from nine studies in five countries identified narrow threshold ranges for DR (FPG: 6.4–6.8 mmol/L and HbA1c: 6.3–6.7%). The conclusions of several longitudinal studies [19–21] also differed; some [19] reported a threshold of hyperglycemia for the increased incidence of DR, supporting the suggested pattern of the association between glycemia and DR, while others [20, 21] did not identify such a threshold.

In the Chinese population, the evidence is still deficient and controversial. One study [22] concluded a continuous relationship between FPG and DR. However, this study used box plots and scatter plots to show the relationship, without dividing the subjects into deciles. To date, only one study [11] from China has analyzed these associations using the methods described above; this study showed threshold values for the prevalence of DR of 7.2 mmol/L for FPG and 6.4% for HbA1c with the method of distributing the data into deciles and 7.8 mmol/L for FPG and 6.8% for HbA1c by ROC curve analysis. However, that study included only participants whose FPG was ≥5.6 mmol/L, and the real glycemic state, including the HbA1c, of the excluded participants (4324 individuals; 53.5% of the total participants screened in that study) and whether the participants received antihyperglycemic medication or not were not described. In our present study, 6 participants with FPG < 5.6 mmol/L had HbA1c ≥ 6.5%, and DR was found in 5 participants with FPG < 5.6 mmol/L. Thus, the exclusion of participants with FPG < 5.6 mmol/L may disguise the associations between DR and glycemic measures. Accordingly, the present study was more representative of the whole population, as we randomly enrolled the residents, regardless of their blood glucose state. Nevertheless, our results corresponded with those of the former study [11] in China, with both showing a higher FPG and a similar HbA1c cutoff in the Chinese population compared with the WHO criteria.

Moreover, these results were marginally different from studies in other regions of Asia. Particularly, the cutoff of FPG by deciles in the Chinese population (7.03–7.2 mmol/L) was much higher than those in other Asian populations (6.2–6.5 mmol/L) [13, 15, 16, 23, 24]. However, in our study, the optimal FPG, determined using ROC curve analysis (6.52 mmol/L), was close to that in the previous Asian studies. Moreover, the cutoff of HbA1c in the Chinese population (6.4%) was higher than that of Japanese studies (5.8–5.9%) [13, 15] and lower than that of a study from Singapore (6.9%) [24], but close to the values reported in Korean studies (6.4–6.9%) dividing the participants into deciles [16, 23]. Further, the HbA1c cutoff determined using ROC curve analysis (5.9%) in our study was similar to the values from the Japanese studies. The discrepancies in the specific glycemic cutoffs even between different Asian populations may suggest a special genetic background of the Chinese population or simply be due to other influences such as the different definitions of DR, different statistical methods used, and differences in the inclusion or exclusion of patients taking diabetes medications.

Because a uniform glycemic cutoff did not exist among the previous studies, we tested the WHO criteria of diagnosing diabetes in this population and found that the specificity of all three measures was high but that they showed relatively low sensitivity for diagnosing DR. These results suggest that, in this population, a proportion of people with DR had a glycemic state below that specified in the current diagnostic criteria. Actually, in this dataset, we found that 15% of the participants with DR had normal plasma glucose levels (FPG < 6.1 mmol/L and 2hPG < 7.8 mmol/L), and none of their characteristics or risk factors differed from the other participants with normal glycemic status. This finding illustrates that factors other than plasma glucose and blood pressure may influence the onset of retinopathy. Similarly, other studies [14, 17] have also reported a high prevalence of retinopathy among people with lower FPG, and the Diabetes Prevention Program found that 8% of people with FPG below diabetic levels had retinopathy [25]. Nevertheless, the significance of retinopathy in patients without diabetes needs further investigation in longitudinal studies.

The present study has some important strengths in that we used a randomized sample of the general population to evaluate the associations of glycemic measures and retinopathy and that it confirmed the threshold effects of FPG and HbA1c for the prevalence of DR. In addition, we found that retinopathy existed in a considerable proportion of individuals with normal plasma glucose, and statistical analysis showed no difference in the baseline characteristic of these individuals compared with those without retinopathy. Longitudinal researches are needed to further investigate these individuals.

Nonetheless, this study also has several limitations. First, patients with known diabetes and on diabetes medication were included in the analysis, leading to possible bias in the distribution of glycemia; however, after excluding those patients, a threshold effect was not detected. This may be ascribed to the relatively small sample size and that the exclusion of these participants destroyed the natural distribution of the glycemic state in the population. Second, the response rate was 66.9%, leading to possible selection bias. Third, this was a cross-sectional study, and we hence cannot conclude whether the onset of DR was associated with the distribution of glycemia or not.

In conclusion, the present study found that, in this sample of the general Chinese population, the prevalence of retinopathy significantly increased in the tenth deciles of FPG and HbA1c, with optimal cutoffs of 7.03 mmol/L and 6.4%, respectively. The current WHO guideline for diagnosing diabetes has high specificity but low sensitivity for detecting DR in this population.

## Figures and Tables

**Table 1:** Characteristics of the participants divided according to the presence of DR (ETDRS scale score ≥ level 20).

	With DR	Without DR	*p*
N	40	3084	
Age (years)	55.6 ± 8.1	47.8 ± 11.8	<0.001
Male (%)	42.5	48	0.487
BMI (kg/m^2^)	25.9 ± 3.8	26.2 ± 3.8	0.635
SBP (mmHg)	140.1 ± 18.6	129.2 ± 17.0	<0.001
DBP (mmHg)	91.2 ± 14.6	85.0 ± 11.6	0.011
FPG (mmol/L)	8.4 ± 2.8	5.9 ± 1.5	<0.001
2hPG (mmol/L)	11.9 ± 7.5	7.2 ± 3.3	<0.001
HbA1c (%)	7.2 ± 1.6	5.6 ± 0.9	<0.001
Fasting insulin (mU/L)	8.9 (4.1, 13.4)	7.4 (4.7, 11.3)	0.398
2 h postload insulin (mU/L)	22.1 (14.5, 45.2)	40.1 (23.1, 67.0)	0.014
ALT (U/L)	21.5 (16.0, 27.5)	20.0 (15.0, 27.0)	0.368
AST (U/L)	20.0 (17.0, 25.0)	21.0 (18.0, 25.0)	0.764
SCR (*µ*mol/L)	52.0 (43.4, 64.5)	58.0 (49.0, 68.4)	0.026
UA (mmol/L)	251.5 (208.0, 297.3)	273.0 (227.0, 331.0)	0.028
TC (mmol/L)	4.8 (4.3, 5.5)	4.8 (4.3, 5.5)	0.939
TG (mmol/L)	1.1 (0.7, 1.9)	1.2 (0.8, 1.9)	0.889
HDL-C (mmol/L)	1.1 (1.0, 1.4)	1.1 (1.0, 1.3)	0.679
LDL-C (mmol/L)	2.7 (2.2, 3.2)	2.8 (2.3, 3.3)	0.477
UACR (mg/g)	24.6 (3.2, 64.6)	5.8 (2.2, 15.1)	0.001

Variables with a normal distribution are presented as the mean ± standard deviation; other variables are presented as the median (interquartile range).

DR, diabetic retinopathy; ETDRS, Early Treatment of Diabetic Retinopathy Study; BMI, body mass index; SBP, systolic blood pressure; DBP, diastolic blood pressure; FPG, fasting plasma glucose; 2hPG, 2-hour postload plasma glucose; HbA1c, glycated hemoglobin A1c; ALT, alanine aminotransferase; AST, aspartate aminotransferase; SCR, serum creatinine; UA, uric acid; TC, total cholesterol; TG, triglycerides; HDL-C, high-density lipoprotein cholesterol; LDL-C, low-density lipoprotein cholesterol; UACR, urine albumin-to-creatinine ratio.

**Table 2:** Performances of glycemic cutoff points derived from different analytic methods and the WHO criteria in detecting DR.

	Cutoff point	Sensitivity	Specificity
Decile distribution			
FPG (mmol/L)	7.03	0.650	0.907
HbA1c (%)	6.4	0.625	0.904
ROC curve analysis			
FPG (mmol/L)	6.52	0.750	0.858
HbA1c (%)	5.9	0.775	0.784
WHO criteria			
FPG (mmol/L)	7.0	0.650	0.905
2hPG (mmol/L)^†^	11.1	0.353	0.917
HbA1c (%)	6.5	0.625	0.995

^†^Analyzed in 2959 participants after excluding those with known diabetes.

WHO, World Health Organization; DR, diabetic retinopathy; FPG, fasting plasma glucose; HbA1c, glycated hemoglobin A1c; ROC, receiver operating characteristics; 2hPG, 2-hour postload plasma glucose.
